# Effects of Yoga Respiratory Practice (*Bhastrika pranayama*) on Anxiety, Affect, and Brain Functional Connectivity and Activity: A Randomized Controlled Trial

**DOI:** 10.3389/fpsyt.2020.00467

**Published:** 2020-05-21

**Authors:** Morgana M. Novaes, Fernanda Palhano-Fontes, Heloisa Onias, Katia C. Andrade, Bruno Lobão-Soares, Tiago Arruda-Sanchez, Elisa H. Kozasa, Danilo F. Santaella, Draulio Barros de Araujo

**Affiliations:** ^1^Brain Institute, Federal University of Rio Grande do Norte (UFRN), Natal, Brazil; ^2^Onofre Lopes University Hospital, Federal University of Rio Grande do Norte (UFRN), Natal, Brazil; ^3^Department of Biophysics and Pharmacology, Federal University of Rio Grande do Norte (UFRN), Natal, Brazil; ^4^Department of Radiology, Medical School, Federal University of Rio de Janeiro, Rio de Janeiro, Brazil; ^5^Hospital Israelita Albert Einstein, São Paulo, Brazil; ^6^Sports Center, University of São Paulo (CEPE-USP), São Paulo, Brazil

**Keywords:** yoga, pranayama, anxiety, affect, emotion regulation, functional MRI, amygdala, insula

## Abstract

*Pranayama* refers to a set of yoga breathing exercises. Recent evidence suggests that the practice of *pranayama* has positive effects on measures of clinical stress and anxiety. This study explored the impact of a *Bhastrika pranayama* training program on emotion processing, anxiety, and affect. We used a randomized controlled trial design with thirty healthy young adults assessed at baseline and after 4 weeks of *pranayama* practices. Two functional magnetic resonance imaging (MRI) protocols were used both at baseline and post-intervention: an emotion task as well as a resting-state acquisition. Our results suggest that *pranayama* significantly decreased states of anxiety and negative affect. The practice of *pranayama* also modulated the activity of brain regions involved in emotional processing, particularly the amygdala, anterior cingulate, anterior insula, and prefrontal cortex. Resting-state functional MRI (fMRI) showed significantly reduced functional connectivity involving the anterior insula and lateral portions of the prefrontal cortex. Correlation analysis revealed that changes in connectivity between the ventrolateral prefrontal cortex and the right anterior insula were associated with changes in anxiety. Although it should be noted that these analyses were preliminary and exploratory, it provides the first evidence that 4 weeks of *B. pranayama* significantly reduce the levels of anxiety and negative affect, and that these changes are associated with the modulation of activity and connectivity in brain areas involved in emotion processing, attention, and awareness. The study was registered at https://www.ensaiosclinicos.gov.br/rg/RBR-2gv5c2/(RBR-2gv5c2).

## Introduction

Yoga is a system of practices with ancestral roots in India (1). It is defined as *Chitta Vritti Nirodhah*—the cessation of the whirlwinds of the mind—which is better understood in contemporary language as a tool to calm the mind (2). The Yoga Sutras of Patañjali systematized it a set of eight practices, also called *Ashtanga* Yoga or Yoga of the eight limbs (1, 2): *yamas* (abstentions), *niyamas* (observances), *asanas* (postures), *prāṇāyāma* (control of breath), *pratyāhāra* (withdrawal of senses), *dhāraṇā* (concentration), *dhyāna* (meditation), and *samādhi* (oneness). The breathing practices are called *prāṇāyāma*, which is a Sanskrit word for *prana* (vital energy) and *ayama* (control). It refers to a series of voluntary controlled breathing exercises that manipulate the respiratory frequency, inhalation (*puraka*), retention (*kumbhaka*), exhalation (*rechaka*), and body locks (*bandhas*) (3).

The practice of pranayama influences many physiological variables. Evidence suggests that its practice produces a positive impact on the cardiorespiratory system (4–7), where slow-paced breathing leads to reduced heart rate and decreased systolic and diastolic blood pressure (8), while fast breathing leads to less robust, but consistent increase in heart rate (9–12). In fact, a previous study observed that the practice of the *Bhastrika pranayama* with low respiratory rate decreased significantly both the systolic and diastolic blood pressure, with a modest decrease in heart rate (10). Furthermore, changes in heart rate variability (HRV) also support the notion that the practice of *pranayama* improves respiratory function and cardiac sympathovagal balance, which are important psycho-physiological stress-related variables (13, 14).

A number of studies support significant positive effects of different yoga practices on anxiety and depression (15, 16) but very few have explored the impact of the practice of *pranayama* on neurophysiological, psychological and psychiatric variables, although evidence suggests improved self-regulation, positive mood, reduced stress, and anxiety (4, 5, 17). A study evaluating the effects of fast and slow pranayama on perceived stress and cardiovascular parameters in young students observed a significant and comparable decrease in the perceived stress scores in both types of pranayama practices, while cardiovascular parameters were changed only after the slow-paced pranayama (18). Furthermore, evidence suggests that yoga programs that include pranayama result in reducing anxiety in humans (19, 20), and a recent feasibility study found evidence of the positive impact of pranayama in patients with treatment-resistant generalized anxiety disorder (21).

It has been hypothesized that the psychobiological mechanism through which pranayama exerts its effects are mediated by the vagus nerve, through interconnections between peripheral sensory organs, the solitary nucleus, thalamus, limbic areas, and the prefrontal cortex (17, 22). Furthermore, it has been suggested that the increase of parasympathetic activity (associated with expiration time) reduces the release of hormones associated with stress (22, 23), and enhances GABA inhibition from the prefrontal cortex and insula to the amygdala, reducing its activity, and the psychological and somatic symptoms associated with stress (24, 25).

Recent studies show that yoga practices, such as meditation, are associated with emotional regulation processes (26, 27). It remains unclear, however, whether these changes occur through top-down or bottom-up strategies. The emotional regulation task used in this trial allowed us to investigate both processes. In addition, several brain regions involved in emotion regulation, as the amygdala, insula, and anterior cingulate cortex (ACC) play an important role in anxiety disorders (28), and previous evidence suggests that anxiety-prone individuals have increased activity in the bilateral amygdala and insula when compared to healthy controls (29). Furthermore, the practice of meditation, including focused on breathing, leads to optimized emotion regulation through increased acceptance and enhanced present-moment awareness (30–33) while impaired emotion regulation has been associated with depression and anxiety (34, 35).

However, the neural basis of the effects of pranayama on anxiety, mood, and emotional regulation, has been less explored. This study aimed at exploring the impact of a 30-day training program of *B. pranayama* on a brain network involved in emotion processing and its association with self-reported changes in affect and anxiety. We used functional magnetic resonance imaging (fMRI) to assess changes in activity and connectivity of brain networks involved with anxiety and emotion processing, and questionnaires to access states of anxiety and affect. Our hypotheses were that pranayama training would: i) decrease anxiety levels; ii) decrease negative affect levels; iii) increase positive affect levels; iv) decrease connectivity within emotion processing brain networks; v) decrease activity in the amygdala; vi) increase activity in parts of the prefrontal cortex, anterior cingulate, and insula, all involved in emotion processing and anxiety.

## Materials and Methods

### Participants

Participants were recruited through word-of-mouth and printed advertisements posted at the Federal University of Rio Grande do Norte campus and community. After contacting the experimenter, participants received information about the goal of the study as well as the exclusion criteria. They were informed that they could be allocated in either a group that involves pranayama practices or in a control group, depending on randomization.

Thirty volunteers were selected according to the following inclusion criteria: i) healthy young adults, between 18 and 40 years of age, naïve to the practice of *pranayama*. Exclusion criteria included: i) MRI contraindication, such as metal parts in the body or pregnancy; ii) chronic rhinitis, with partial or complete obstruction of one or both nostrils; iii) frequent use of bronchodilator; iv) regular use of beta-blocker, stimulants or any other substance that interferes with cardiovascular activity; and v) current diagnosis or history of neurological or psychiatric disorders. The Ethics and Research Committee of the Federal University of Rio Grande do Norte approved the study (protocol #579.226), and all subjects provided written informed consent prior to their participation in the study. This study was registered at http://www.ensaiosclinicos.gov.br/rg/RBR-2gv5c2/(RBR-2gv5c2).

### Experimental Protocol

**Figure 1:**
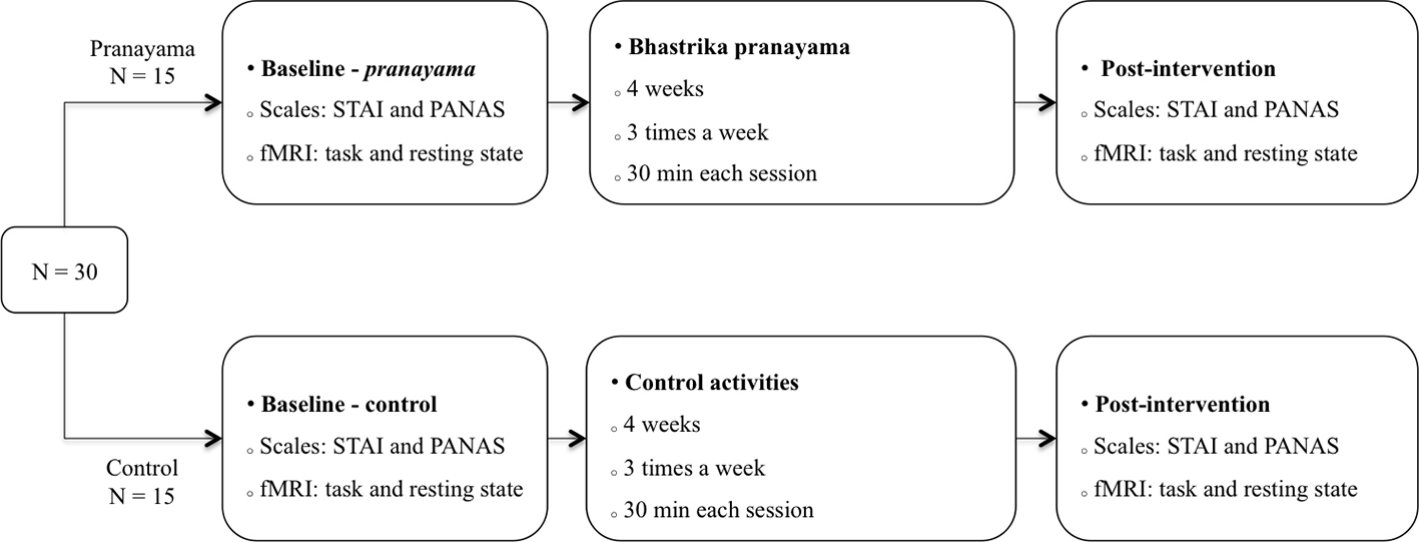
Experimental protocol. Assessments at baseline and after intervention included measurements of anxiety (STAI), affect (PANAS), and fMRI (emotion processing task and resting-state protocols). STAI, State-Trait Anxiety Inventory; PANAS, Positive Affect and Negative Affect Scale; fMRI, functional magnetic resonance imaging.

This study used a randomized controlled design with two parallel arms, and it was conducted in accordance with the consolidated standards of reporting trials (CONSORT) statement (36). Subjects were allocated in blocks of (4:4) to either a pranayama training group or to a control group, using permuted block randomization (http://www.randomization.com). Given the nature of the training, participants and researchers were not blinded. Nevertheless, all the analyses were conducted blindly. In addition, to ensure motivation, those in the control group interested in pranayama were put in a waiting list to receive the training after the study. **Figure 1** shows the experimental protocol used in the study. Participants from both groups (*pranayama* and control) were assessed at baseline and right after a 30-days training program. Both assessments included fMRI and psychometric trait-state measures of affect and anxiety.

The level of anxiety was assessed by the State-Trait Anxiety Inventory (STAI), which has been translated and validated to Brazilian Portuguese (37). We also used the Positive Affect and Negative Affect Scale (PANAS) to assess positive affect (PANAS-P), such as well-being, enthusiasm, inspiration and determination, and negative affect (PANAS-N), aimed at dimensions such as fear, nervousness, and disturbance. We used the adapted and validated PANAS to Brazilian Portuguese (38, 39). Both scales were applied to assess state and trait characteristics. fMRI assessments included an emotional regulation task and a resting state (rs-fMRI) protocol, described below in detail.

### The Training Program

The practice of the *B. pranayama* is not easy for inexperienced Yoga practitioners. Therefore, during the initial 5 days of training, participants were guided for 30 min a day for the correct practice of *B. pranayama*. An instructor was present during these encounters and followed a specific sequence of daily steps designed to guide participants into the correct practice. Each training session had only four participants at a time to allow careful and individualized training.

This initial period was followed by 4 weeks of regular *B. pranayama* practice. In order to assure a volume of practices similar to previous studies from our group (40) and to the ones focused on mindfulness meditation (41), our study was designed with five practices a week, for 4 weeks. For 3 days a week, 30 min per day, participants gathered and practice together with an instructor in supervised training classes. Besides in-person meetings, subjects were instructed to practice at home for at least two more days a week. The control group also gathered with the same frequency and duration but performed ludic cognitive activities such as crosswords, puzzles, domino, checkers, and card games, also in the presence of an instructor. Participants performed one of these activities at each encounter and were asked to alternate between activities to ascertain that each game was practiced at least one to two times during the 12 scheduled meetings. Subjects in the control group were also instructed to practice at home.

Each *B. pranayama* session started with a brief 2-min savasana (relaxation), followed by 5 min of asanas (*pavanamuktasana*, *sukhasana*, *gomukhasana*, *paschimotanasana*, and *vakrasana*) applied solely to prepare the body for the practice of pranayama, as described in Patañjali's Yoga Sutra. Following this short preparation, the *B. pranayama* was performed continuously for 25 min. Sessions ended with another brief savasana (2 min).

The practice of the *B. pranayama* used in this study followed the description of Swami Kuvalayananda (13), according to whom, each round of the practice is composed by a set of fast breathing (kapalabhati) followed by a slow inspiration through the right nostril, a comfortable apnea done with the three bandhas (*mula*, *jalandhara*, and *uddiyana*) and a slow expiration through the left nostril (Surya bedhana). The relation inspiration:apnea:expiration was set according to individual comfort, varying from 1:1:2 to 1:2:2; 1:3:2, or 1:4:2—apnea never exceeded four times the inspiration time, and expiration was set to constantly correspond to twice the inspiration time.

Each *kapalabhati* consists of a series of 30 rapid self-paced expirations generated by contractions of the *rectus abdominis* muscle. Contrary to natural breathing, the *kapalabhati* inspiration is passive while expiration is active. One-cycle of *Surya bedhana* is composed of a slow inspiration through the right nostril, followed by apnea and by a longer, yet comfortable expiration through the left nostril. The suggested ratio between inspiration:apnea:expiration followed the traditional description of 1:4:2. Inspiration, apnea, and expiration times were set individually, according to one's capacity and comfort. Therefore, when the 1:4:2 ratio was felt uncomfortable, volunteers were instructed to decrease apnea duration to one of the following alternative ratios: 1:3:2; 1:2:2, or 1:1:2. During periods of apnea, practitioners were also instructed to execute three *bandhas*, also called locks: *jalandhara bandha*, *uddyiana bandha*, and *mula bandha*. *Jalandhara bandha* is attained by pressing the chin against the jugular notch, with both nostrils closed with the fingers, *uddyiana bandha* by a chest expansion after *jalandhara bandha*, followed by perineum contraction, called *mula* bandha. Participants were instructed to perform 30 cycles of the *B. pranayama* at each encounter.

### Magnetic Resonance Imaging

#### Imaging Acquisition

Images were acquired in a 1.5-T MRI scanner (HDxt, General Electric, USA). Functional MRI datasets were acquired with the following EPI sequence parameters: repetition time (TR) = 2000 ms; echo time (TE) = 35 ms; flip angle = 60˚; field of view (FOV) = 24 cm; matrix = 64 × 64; slice thickness = 3 mm; gap = 0.3 mm; number of slices = 35; volumes = 165 (emotion processing) and 213 (resting state). High-resolution T1-weighted images were acquired with the following FSPGR BRAVO sequence: TR = 12.7 ms; TE = 5.3 ms; flip angle = 60˚; FOV = 24 cm; matrix = 320 × 320; slice thickness = 1.0 mm; number of slices = 128.

#### Emotion Processing Protocol

All subjects had normal or corrected to normal vision. Immediately before scanning, they received a training session to ensure task compliance, using another set of images for stimuli. During the fMRI emotion processing task, programmed using Psychopy v.1.79 (42), subjects viewed a series of images with different emotion valence (neutral or negative) and were asked to rate the emotional impact of these images using a 5-point Likert scale (very negative, negative, neutral, positive, very positive). Responses were recorded *via* a five-button fiber optic response pad (Current Designs, Philadelphia, USA).

We used pictures from the International Affective Picture System (IAPS) (43) dataset, from which we selected 72 pictures with negative valence and 36 with neutral valence. Images were classified using the valence and arousal scores: negative images had low valence and high arousal scores and neutral images had medium valence and low arousal scores. During negative images presentation, participants were instructed to either observe passively the image or to try to reappraise it, depending on the instruction displayed previous to the image on the screen. In this context, reappraisal describes the attempt to attribute a new meaning to the arousing stimulus in order to reduce its emotional impact (44, 45). A list of possible cognitive reappraisal strategies was presented to the participants beforehand, such as thinking that the pictures were not of a real scene, imagining that the image was from a movie, or imagining a happy ending to the situation depicted on the scene. In addition, we explicitly asked participants to avoid closing their eyes or distracting themselves from the picture. Three conditions served to form 3 fMRI predictors: i) passively looking at neutral pictures (NEU); ii) passively looking at negative pictures (NEG); iii) reappraisal of negative pictures (REAP).

During each fMRI session, subjects were scanned in three separate runs (~5.5 min each), comprising 18 trials, 6 for each condition (NEU, NEG, REAP). Each trial lasted 18 s: 2 s for instructions (LOOK or REAPPRAISE), followed by 6 s of picture presentation, 6 s for the button response, and 4 s of a fixation cross presented at the center of the screen (**Figure S1**).

#### fMRI Emotion Processing Analysis

The fMRI emotion processing task was analyzed using SPM12 (Statistical Parametric Mapping, UK). The first three volumes were discarded to allow for T1 stabilization. Preprocessing steps included head motion correction, slice timing correction, spatial smoothing [8-mm full width at half maximum (FWHM) Gaussian kernel], and a high-pass filter (128 s). EPI images were coregistered to each subject's anatomical scan, normalized into standardized MNI space and resampled to voxels of 2 mm^3^. Serial autocorrelations were accounted for by a first-order autoregressive model (AR1). In addition, motion artifact was examined using the Artifact Detection Toolbox (ART) and volumes with a global signal deviation superior to three SD from the mean signal or in which the difference in frame displacement (FD, a composite measure of movement) between two consecutive volumes exceeded 1 mm, were considered as outlier volumes. The entire run was excluded if more than 15% of all volumes behaved as outliers.

A first-level fixed-effects model was used for each subject and each session. Five regressors corresponding to the three conditions (NEU, NEG, REAP), instruction, and rating periods were modeled using a boxcar function convolved with a canonical hemodynamic response function. Periods of fixation cross were defined as the baseline. The model also included six motion parameters and outlier volumes as regressors of no interest. Images contrasts were calculated using t-statistics for: i) NEG, ii) REAP, iii) NEG vs. REAP.

The effect of *pranayama* was inspected in the following region of interest (ROI), previously implicated in emotional processing (46, 47): ACC, amygdala, anterior insula, orbitofrontal cortex (OFC), dorsolateral prefrontal cortex (dlPFC), dorsomedial prefrontal cortex (dmPFC), ventrolateral prefrontal cortex (vlPFC), and ventromedial prefrontal cortex (vmPFC). With the exception of vmPFC (48), and the anterior insula (49) all other ROI were obtained directly from the WFU PickAtlas (50) in SPM12 (**Figure S2**). Mean β-values for each ROI were extracted from each subject's contrast using MarsBaR (SPM12).

#### rs-fMRI Data Acquisition **and** Analysis

The resting-state fMRI acquisition lasted for ~7.1 min, and subjects were instructed to lie down with their eyes closed, avoiding to move or fall asleep. Resting-state data were analyzed using a functional connectivity (fc) toolbox (CONN, https://www.nitrc.org/projects/conn). The first three volumes were discarded to allow for T1 equilibrium. Preprocessing steps included head motion correction, slice timing correction, and spatial smoothing (8-mm FWHM Gaussian kernel). Functional and structural images were normalized to the MNI template. Quality control included motion artifacts inspection with ART. Outlier volumes were considered when the global signal deviated more than three standard deviations (SD) from the mean signal or when the difference in FD between two consecutive volumes exceeded 0.5 mm. Denoising step included: CompCor method (to remove physiological and other sources of noise); use of six motion parameters and its derivatives as regressors of no interest; scrubbing and a band-pass filter (0.01–0.1Hz).

We explored the same ROI used in the emotion processing task. A 16 × 16 correlation matrix was generated by computing Pearson's coefficients between the averaged time-series for every pair of ROI.

### Statistical Analysis

Demographic variables were compared using the independent Student's t-test when continuous, or chi-square test when categorical. To evaluate the effects of the *B. pranayama* on anxiety and affect, and β-values changes, we used a repeated-measures ANOVA (RM-ANOVA) with two factors: intervention (pranayama × control) and time (baseline × post-intervention). In cases where we found significant interactions, within groups *post hoc* analyses were conducted using the dependent Student's t-test. To further investigate the relationship between fMRI changes and the effects of *B. pranayama*, Pearson's correlation analyses were conducted to explore associations between changes in β-values from baseline to post-intervention (Δβ= βafter–βbefore) and changes in STAI and PANAS scores from baseline to post-intervention. Also, correlation coefficients were estimated only when significant interaction was found. We set the statistical threshold at p < 0.05 and used Cohen's d to estimate effect sizes. Statistical analyses were performed using GraphPad Prism version 7.00 (GraphPad Software, La Jolla CA, USA). Since the trial adheres to the CONSORT statement, between-group baseline differences of primary outcomes should not be reported (51).

## Results

The participants recruited for our study were healthy young adults (25.1 ± 4.3 years old; 15 women), mostly university students. There were no significant between-groups differences (control × pranayama) with respect to age (t_28_ = 1.41; p = 0.16), gender (χ^2^ = 0.13; p = 0.71), years of education (χ^2^ = 2.91; p = 0.23), and household income (χ^2^ = 0.57; p = 0.90). Detailed demographic and clinical characteristics are presented in **Table 1**. **Figure S3** shows the CONSORT flow diagram for the trial.

**Table 1:** Socio-demographic and clinical characteristics.

	Pranayama	Control	p-value
Gender (n)			0.71
Male	7	8	
Female	8	7	
Age—years (mean ± SD)	24 ± 4.47	26.2 ± 4.05	0.16
Education—years (n)			0.23
9–11 years	4	5	
12–16 years	10	6	
17 or more years	1	4	
Household income (n)			0.90
<2 minimum wage	6	5	
2–5 min. wage	4	3	
6–10 min. wage	3	4	
11 or more min. wage	2	3	
STAI State (mean ± SD)	37.92 ± 4.09	38 ± 8.32	
STAI Trait (mean ± SD)	42.57 ± 6.47	37.80 ± 8.75	
PANAS-P State (mean ± SD)	31.50 ± 3.44	31.60 ± 6.14	
PANAS-P Trait (mean ± SD)	32.64 ± 5.11	35.40 ± 5.88	
PANAS-N State (mean ± SD)	14.79 ± 3.02	13.15 ± 2.51	
PANAS-N Trait (mean ± SD)	18.07 ± 3.83	16.67 ± 5.73	

STAI, State-Trait Anxiety Inventory; PANAS, Positive Affect and Negative Affect Scale.

### Effects of *B. pranayama* on Anxiety and Affect

One subject from the *pranayama* group did not attend the second evaluation due to health issues. Boxplots were used for outlier identification (values above or below 1.5 times the interquartile range), which resulted in the exclusion of one subject from the *pranayama* group in the STAI and two from the control group in the PANAS-N (**Figure S4**).

**Figure 2:**
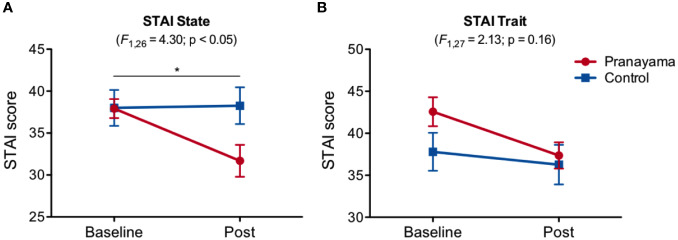
Effects of *Bhastrika pranayama* on anxiety. STAI (state and trait) scores in both groups (pranayama and control) at baseline and after the intervention. **(A)** A significant interaction (intervention × time) was observed in state anxiety (*F*_1,26_ = 4.30; p = 0.048) with significantly decreased scores within the *pranayama* group (t_12_ = 3.01; p = 0.01). **(B)** No significant interaction was observed in trait anxiety. Graphs depict mean values and standard error of the mean. *p < 0.05.

**Figure 2** shows changes in the STAI state and trait scores for both groups (*pranayama* and control) from baseline to post-intervention. We observed an interaction effect (intervention × time) in state of anxiety (*F*_1,26_ = 4.30; p = 0.048, Cohen's d = 0.81), with significant decreased levels in the *pranayama* group after intervention (t_12_ = 3.01; p = 0.01; **Figure 2A**). No significant interaction was observed in trait anxiety (*F*_1,27_ = 2.13; p = 0.16; **Figure 2B**).

**Figure 3:**
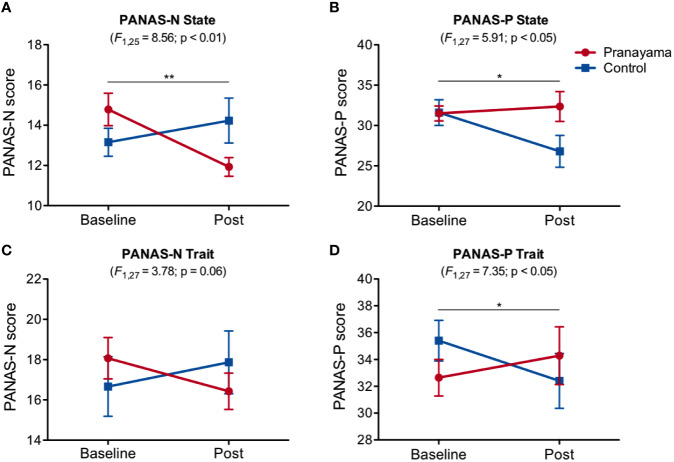
Effects of *Bhastrika pranayama* on affect. PANAS (state and trait), positive (PANAS-P), and negative (PANAS-N) scores for both groups (*pranayam*a and control) at baseline and after the intervention. Significant interactions (intervention × time) were observed in state affect, both negative **(A)** F_1.25_ = 8.56; p = 0.007, Cohen's d = 1.17) and positive **(B)** F_1,27_ = 5.91; p = 0.02, Cohen's d = 0.93), with a significant decrease in negative affect within the *pranayama* group (t_13_ = 3.43; p = 0.004). For trait PANAS, a trend for PANAS-N **(C)** F1,27 = 3.78; p = 0.06; Cohen's d = 0.78; was found. A significant interaction effect in PANAS-P **(D)** F1,27 = 7.35; p = 0.012, Cohen's d = 1.04; Graphs depict mean values and standard error of the mean. **p < 0.01, *p < 0.05.

**Figure 3** shows the observed changes in PANAS state and trait scores for both groups before and after intervention. We observed significant interaction effect (intervention × time) in the state of negative affect (state PANAS-N, F_1.25_ = 8.56; p = 0.007, Cohen's d = 1.17; **Figure 3A**), with a significant decrease within the *pranayama* group (t_13_ = 3.43; p = 0.004; **Figure 3A**). States of positive affect were also significantly changed, with a significant interaction (intervention × time) effect (PANAS-P; F_1,27_ = 5.91; p = 0.02, Cohen's d = 0.93; **Figure 3B**). For trait PANAS, we found a significant interaction (intervention × time) effect in PANAS-P (F_1,27_ = 7.35; p = 0.012, Cohen's d = 1.04; **Figure 3D**), and a trend for PANAS-N (F_1,27_ = 3.78; p = 0.06; Cohen's d = 0.78; **Figure 3C**).

### Effects of *B. pranayama* on fMRI

fMRI analysis was conducted in 13 subjects from each group. One subject from the *pranayama* group did not attend the second fMRI session due to health issues and three subjects were excluded due to excessive head motion artifact, two from the control group (C8 and C12) and one from *pranayama* group (P8).

### Emotion Processing Task—Behavioral Results

Scores attributed to the emotional impact of each image were analyzed using an ANOVA with two within-subjects factors: condition (NEU, NEG, REAP) and time (baseline and post-intervention), and one-factor between-subjects: intervention (pranayama and control). We observed a main effect for condition (F_2,24_ = 96.72; p < 0.0001) and *post hoc* comparisons, corrected by the Sidak test, showed significant differences between conditions NEG × NEU (p < 0.0001) and NEG × REAP (p < 0.0001). There were no significant differences in REAP × NEU condition (p = 0.73). No effects of time, intervention or interactions between them were found.

### Emotion Processing Task: fMRI Results

Effects of the *B. pranayama* were analyzed with an RM-ANOVA with a time factor (baseline and post-intervention) and an intervention factor (control and *pranayama*) in each condition (NEG, REAP, and NEG > REAP).

**Figure 4:**
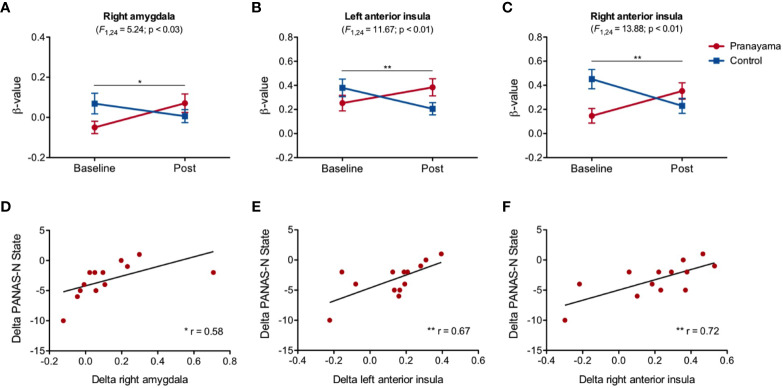
Effects of the intervention in the NEG condition. **(A)** Significant interaction effect(intervention × time) in the right amygdala (F_1,24_ = 5.24; p = 0.03; Cohen's d= 0.93); **(B)** left anterior insula (F_1,24_ = 11.67, p = 0.002; Cohen's d = 1.39); **(C)** right anterior insula (F_1,24_ = 13.88; p = 0.001; Cohen's d = 1.52). Beta values and standard error of the mean for each group (*pranayama* and control) before and after intervention. Correlation between changes in β-values and PANAS scores. Significant Pearson's correlation analysis between change in state negative affect (PANAS-N) and changes in the activity of **(D)** the right amygdala (r=0.59, p = 0.034); **(E)** left anterior insula (r = 0.67, p = 0.012); **(F)** right anterior insula (r = 0.72, p = 0.005), in the *pranayama* group. Correlation values are represented as changes in individual β-values (after-before) and changes in individual scale scores (after-before). *p < 0.05, **p < 0.01.

**Figure 4** shows the results in the NEG condition (**Figure S5** shows the main effect for this condition). We observed a significant interaction in the right amygdala (F_1,24_ = 5.24; p = 0.03, Cohen's d = 0.93; **Figure 4A**), the left anterior insula (F_1,24_ = 11.67, p = 0.002, Cohen's d = 1.39; **Figure 4B**), and in the right anterior insula (F_1,24_ = 13.88; p = 0.001, Cohen's d = 1.52; **Figure 4C**). Within-group analysis in the *pranayama* group showed significant increased activity after intervention in bilateral anterior insula (right: t_12_ = 3.01; p = 0.011; left: t_12_ = 2.62; p = 0.023).

Correlation analysis revealed a significant association between state of negative affect and changes in the activity of the right amygdala (r = 0.59, p = 0.034; **Figure 4D**), left anterior insula (r = 0.67; p = 0.012; **Figure 4E**), and right anterior insula (r = 0.72; p = 0.005; **Figure 4F**). These associations revealed that subjects in the *pranayama* group with increased activity in these areas had the least decreased state of negative affect. We found no significant correlation between BOLD signal changes and behavior on the emotional regulation task.

**Figure 5:**
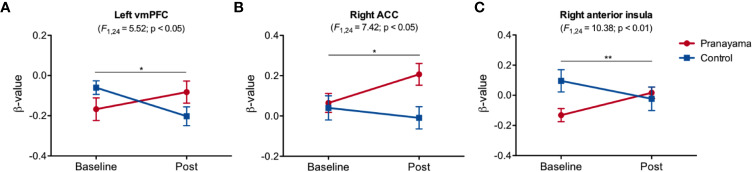
Effects of the intervention in the reappraisal condition. Significant interaction was found in **(A)** the left vmPFC (F_1,24_ = 5.52, p = 0.027, Cohen's d = 0.95); **(B)** right ACC (F_1,24_ = 7.42, p = 0.012, Cohen's d = 1.11), with significant increased activity in the *pranayama* group (t_12_ = 2.37; p = 0.035); **(C)** right anterior insula (F1,24 = 10.38; p = 0.003, Cohen's d = 1.31). Figures show mean beta values and standard error of the mean for each group (pranayama and control) before and after the intervention. Effects of the intervention in the NEG-REAP condition. *p < 0.05, **p < 0.01.

**Figure 5** presents the results for the REAP condition (**Figure S6** shows the main effect for this condition). We observed significant interaction in the left vmPFC (F_1,24_ = 5.52; p = 0.027; Cohen's d = 0.95; **Figure 5A**) and right ACC (F_1,24_ = 7.42, p = 0.012; Cohen's d = 1.11; **Figure 5B**), with significant increase within the *pranayama* group (t_12_ = 2.37; p = 0.035). **Figure 5C** shows significant interaction in the right anterior insula (F_1,24_ = 10.38; p = 0.004, Cohen's d = 1.31) with respect to the NEG-REAP condition.

### Resting-State fMRI

**Figure 6:**
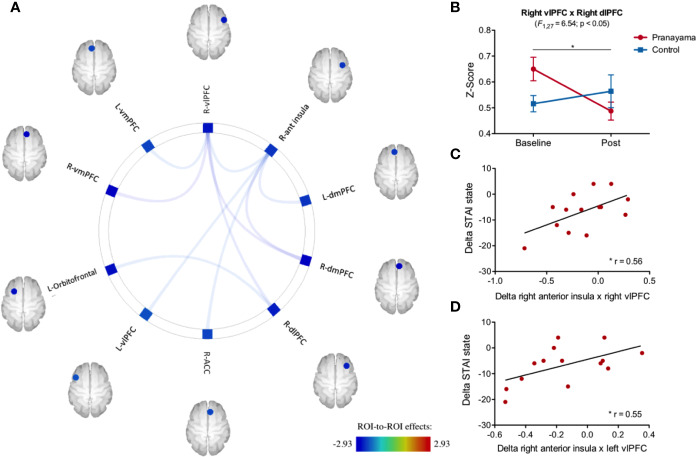
Impact of intervention on functional connectivity and correlation with STAI-s scores. **(A)** Significant interaction effect in the 16 ROI analyzed that are related to emotional processing. **(B)** Significant connectivity interaction effect (intervention × time) between the right vlPFC and the right dlPFC (F_1,27_ = 6.54; p =0.01) with a significant decrease within the *pranayama* group (t_13__=_ 3.97; p = 0.001). **(C)** Pearson's correlation between changes in STAI-s and fc changes between the right anterior insula and right vlPFC (r = 0.56, p = 0.03); and **(D)** Pearson's correlation of changes between the right anterior insula and left vlPFC (r = 0.55, p = 0.03). *p < 0.05.

**Figure 6A** shows the effects of the intervention on fc. The right anterior insula and the right vlPFC were the two regions with the most consistent fc changes both with other ROIs and also with each other. The anterior insula showed significant fc changes with the following areas: right vlPFC (F_1,27_ = 4.72; p = 0.03; Cohen's d = 0.83), left vlPFC (F_1,27_ = 4.73; p = 0.03; Cohen's d = 0.83), right dmPFC (F_1,27_ = 7.07; p = 0.01; Cohen's d = 1.02), left dmPFC (F_1,27_ = 5.17; p = 0.03, Cohen's d = 0.87), and right ACC (F_1,27_ = 4.47; p = 0.04, Cohen's d = 0.86). We observed significant interaction (intervention × time) fc between the vlPFC and the following areas: right vmPFC (F_1,27_ = 8.00; p = 0.008; Cohen's d = 1.08), left vmPFC (F_1,27_ = 4.82; p = 0.003; Cohen's d = 0.84), right dlPFC (F_1,27_ = 6.54; p = 0.01; Cohen's d = 0.98), and right dmPFC (F_1,27_ = 8.57; p = 0.006; Cohen's d = 1.12).

RM-ANOVA analysis showed a significant interaction effect (intervention × time) between the right vlPFC and the right dlPFC (F_1,27_ = 6.54; p = 0.01; Cohen's d = 1.04; **Figure 6B**). *Post hoc* analysis within the *pranayama* group showed significant decreased connectivity between right vlPFC and right dlPFC (t_13_ = 3.97; p = 0.001; **Figure 6B**).

Correlation analysis suggests an association within the *pranayama* group between changes in the state anxiety and changes in connectivity between the right anterior insula and bilateral vlPFC (right: r = 0.56; p = 0.03; left: r = 0.55; p = 0.03), where volunteers with the greater reduction in connectivity had the best outcomes with respect to reduced state of anxiety (STAI-state) (**Figures 6C, D**).

## Discussion

One month of *B. pranayama* training led to significant changes in affect and anxiety, which were associated with changes in activity and connectivity of a few brain areas involved in emotion processing. This exploratory study yielded the following main results: changes in PANAS and STAI scores suggest significantly decreased levels of state of negative affect and anxiety, increased positive affect and fMRI changes suggesting the involvement of the amygdala, anterior insula, ACC, vmPFC, vlPFC, and dlPFC. Although not pathological, our subjects presented measurable levels of anxiety, corresponding to a representative sample of our society, particularly of university students (52). Our results suggest that anxiety levels were significantly reduced, which might encourage the future exploration of the anxiolytic effects of pranayama in a clinical population (21).

Reduced anxiety and changes in affect as an effect of *pranayama* have been observed previously (4, 5, 17, 53), even after a single session of alternate yoga breathing (7). Previous studies report that 3 months of the practice of the *Anuloma-Viloma* (alternating nostril breathing) *pranayama* reduced levels of anxiety (5), as well as after 6 weeks of *Sudarshan Kriya* (4) or 6 months of slow breathing training (17). It has been hypothesized that reduced levels of anxiety are related to change in sympathovagal balance. In fact, stress reduction observed after a yoga breathing training (22, 23) was associated with the predominance of parasympathetic activity found after the practice (13, 54).

In our study, fMRI changes while passive looking at negative images suggest a significant interaction effect in the right amygdala and bilateral insula. Furthermore, individuals with greater increased activity in the amygdala and insula presented less prominent reduced negative affect. Our results are supported by previous evidence suggesting that anxiety-prone individuals have significantly increased activity in the bilateral amygdala and insula when compared to a control population (55).

The amygdala has been the most cited brain region in studies related to emotion processing (56). This structure is part of the limbic system and has been particularly associated with negative emotions (56). fMRI studies in humans have linked increased amygdala responses to emotion-laden stimuli, particularly of fear (57), and bilateral lesions to the amygdala lead to the deterioration of fear recognition and expression (58). There is evidence suggesting functional differences between the right and left amygdala. For instance, electrical stimulation to the right amygdala was related to arousal of negative emotions, while positive and negative emotions were induced when stimulation was applied to the left amygdala (59). We observed changes in the right amygdala, and damage to this area has also been linked to impaired overall autonomic response, such as skin conductance during highly arousal emotional stimulation (60). We found that changes in the amygdala activity were correlated with changes in negative affect. Likewise, it has been observed that positive affect influences amygdala activity (61), and that amygdala activity correlates positively with increased negative affect (62).

The awareness and the emotional impact of a stimulus may be modulated by top-down control mechanisms, such as by reappraisal. In such, cognitive strategies are deliberately used to lessen the impact and emotional response to a given stimulus (63). Evidence suggests that these processes are associated with changes in the activity in prefrontal regions, as well as in the insula and ACC (61, 64, 65). In fact, our results are supportive of the involvement of the ACC during the reappraisal task.

Different models of emotion reappraisal stand to the idea that the attenuation of the impact from negative stimuli would be in part due to a down-regulation of amygdala activity by regions in the prefrontal cortex (66, 67). Emotion reappraisal tasks appear to recruit a network of brain areas involving the prefrontal cortex, particularly of its medial portion but also including vlPFC and dLPFC (46, 68). During reappraisal, we found significant interaction in the prefrontal cortex (vmPFC), which has been implicated in fear processing and a critical brain structure involved in the regulation of amygdala activity (69).

Studies suggest that emotion regulation is also influenced by attention and awareness (70, 71), and the emotional impact of a given stimulus is driven by changes in the activity of the insula, ACC and amygdala (61, 65). In line with this hypothesis, it has been suggested that the practice of meditation is associated with decreased activity in the amygdala in response to emotional stimuli (26, 72–74), besides suggesting the influence of meditation particularly in the insula, ACC, and thalamus (75). Therefore, bottom-up models of emotion regulation seem to better fit the observed brain changes related to contemplative practices, such as meditation and pranayama (76–79).

Functional neuroimaging studies give support to the notion that the insula is an important connection between emotional experiences and the autonomic nervous system (80–82) as anterior insula activity has been predictive of levels of anxiety and trait of interoception (80), besides response to noxious stimuli (83). Together, the insula and ACC form the salience network, with extensive connections to various parts of the brain including the limbic system (84, 85). The salience network has been associated with the awareness of stimuli, and in fact, the anterior insula has been implicated in emotion recognition (86). Therefore, changes observed in the ACC and anterior insula may not necessarily reflect the regulatory process *per se*, but signal differences in the perceived stimuli salience, or awareness (85). Furthermore, there is recent evidence that interoceptive ability can be enhanced by different meditation practices (87, 88) and that such effect is accompanied by structural and functional brain changes. For example, experienced meditators have increased the cortical thickness of the right anterior insula when compared to non-meditators (89), and 8 weeks of mindfulness meditation practice has been related to increased recruitment of areas related to visceral representation, including the right insula, right ACC, vmPFC, and vlPFC (90).

Adding to this hypothesis are the significant changes we observed in the ACC during the emotion regulation portion of the fMRI task. Besides being part of the salience network, the ACC has been implicated in a number of processes of emotion and reasoning, including the reassessment of emotional stimuli (66, 91). The dorsal portion connects primarily to the prefrontal cortex and has been related to executive functions, such as conflict resolution and decision making (84), while subgenual ACC is strongly connected to the limbic system, and its relation to emotion processing goes back to the first models of emotion (92).

During the resting-state condition, we observed significantly reduced functional connectivity between the vlPFC and dlPFC after training, and between bilateral vlPFC and the right anterior insula which was correlated with the observed reduced anxiety. Lateral portions of the PFC have been involved with the regulation of affect and emotion, and cognitive reappraisal tasks consistently recruit PFC regions including the dlPFC and vlPFC, which was found to be inversely correlated with the arousal of the negative emotion (62, 93). While lesions to the vlPFC have been related to heighten negative emotion in monkeys (94), recent human studies suggest the role of the amygdala and vlPFC activity as a predictor of anxiety in young adults (95), and that greater functional coupling between vlPFC and the amygdala is associated with emotion regulation success (96).

Previous studies suggest that the vlPFC is also involved in a number of different tasks that demand cognitive control. Neuroimaging studies support that the vlPFC is particularly important for selective attention either toward goal-relevant information or inhibiting irrelevant information (97). As such, increased activity in the vlPFC is found in response inhibition task (98), semantic processing (99), and during classification tasks (100), just to name a few. It is therefore not clear whether the functional role of the vlPFC is specific to emotion processing, or if it plays a more general role of executive control (99). It should be noted that the changes observed in lateral PFC were observed during the rest condition and not during the performance of a task. It is curious therefore to observe changes in coupling between areas in the brain associated with emotion processing in particular, and selective attention in general, even in the absence of an actual emotion regulation task.

It is also important to point out the major limitations and caveats of the study. First, the lack of adequate assessment of the autonomic nervous system hampers the direct association between changes in anxiety and affect with potential autonomic markers, such as heart rate or interoceptive attention assessment. Individuals were instructed to practice at home, however, we did not control these practices. Therefore, particular variations are expected due to differences related to home practices. The study was designed to assess changes after a single short 30 days intervention against an active control condition. It does not allow the conclusion of how specific the intervention with pranayama really is with respect to other contemplative practices. Therefore, we cannot be absolutely sure that the observed effects were due to pranayama alone, since asanas and savasana also were performed during the sessions, although briefly. The study was conducted in a small number of volunteers, composed mostly of young healthy well-educated individuals, and therefore does not allow the extension of the results to individuals with anxiety disorders or other groups of individuals. It should be noted that these analyses were preliminary and exploratory. Due to sample size limitations and the exploratory nature of this study, for these analyses, we chose not to correct for multiple comparisons, thus the results should be taken with caution and further investigation is recommended.

This study provides the first preliminary evidence that 4 weeks of *B. pranayama* reduced anxiety and increase positive affect, and that these changes are associated with the activity and connectivity of a brain network involved in emotion processing, particularly the amygdala, anterior cingulate, anterior insula, and the prefrontal cortex. Resting-state fMRI revealed significantly reduced functional connectivity particularly involving the anterior insula and lateral portions of the prefrontal cortex which participate in awareness and attention.

## Data Availability Statement

The datasets generated for this study are available on request to the corresponding author.

## Ethics Statement

The studies involving human participants were reviewed and approved by Ethics and Research Committee of the Federal University of Rio Grande do Norte (#579.226). The patients/participants provided their written informed consent to participate in this study.

## Author Contributions

MN, BL-S, FP-F, HO, KA, DS, and DA designed the experiments. MN, FP-F, HO, KA, and DA implemented the protocol. MN and FP-F collected experimental data. MN, BL-S, FP-F, HO, and KA carried out statistical analysis. MN, FP-F, HO, and DA prepared the figures. MN, FP-F, DA, TA-S, EK, BL-S, and DS interpreted and discussed the results. MN, FP-F, and DA prepared the manuscript. MN, FP-F, DA, HO, TA-S, EK, BL-S, and DS revised the manuscript.

## Conflict of Interest

The authors declare that the research was conducted in the absence of any commercial or financial relationships that could be construed as a potential conflict of interest.
