# Supplementary material for: Effects of Yoga Respiratory Practice (Bhastrika pranayama) on Anxiety, Affect, and Brain Functional Connectivity and Activity: A Randomized Controlled Trial
Source: Front Psychiatry. 2020 May 21;11:467. doi: 10.3389/fpsyt.2020.00467 (PMC7253694; doi:10.3389/fpsyt.2020.00467)
Supplement: Supplementary file 1 [file Presentation_1.pdf]

Effects of yoga respiratory practice (*bhastrika pranayama*) on anxiety, affect and brain functional connectivity and activity: a randomized controlled trial

Novaes, et al.

Supplementary material

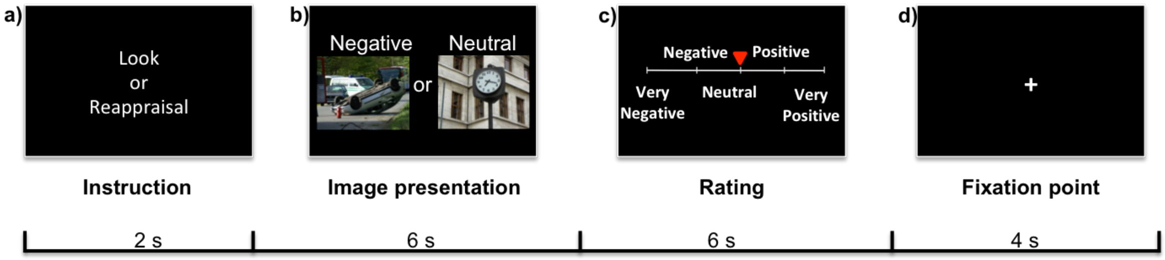

Figure S1. Schematic representation of the emotional regulation protocol. (A) The protocol begins with an instruction (LOOK AT THE PICTURE OR MAKE POSITIVE) lasting two seconds. (B) An image (NEUTRAL OR NEGATIVE) appears for six seconds. (C) A five-point scale (Very Negative, Negative, Neutral, Positive or Very Positive) is presented, and participants have six seconds to rate the emotional impact of the presented image. (D) A fixation point is shown for four seconds. This block (A+B+C+D) is repeated 18 times, six times for each condition (**look at neutral** images, **look at negative** images, make a negative image positive - **reappraisal**). Each run lasted 5 min and participants performed three runs in each fMRI session. Adapted from Ochsner, *et al.,* 2002 (K. Ochsner, Bunge, Gross, & Gabrieli, 2002).

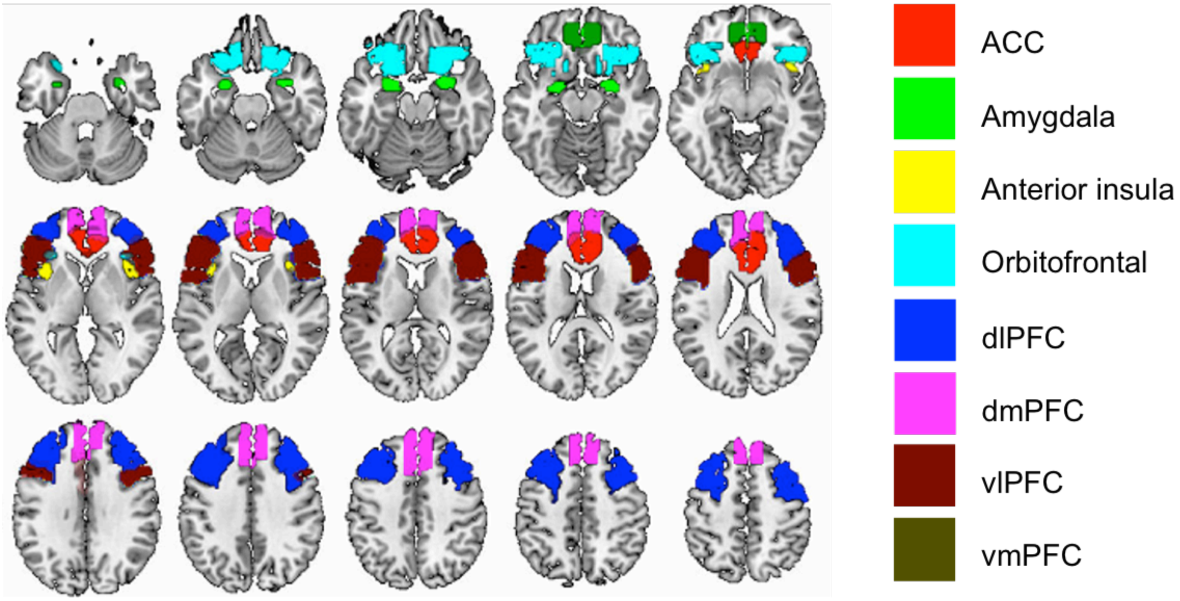

Figure S2. ROI used for rs-fMRI analysis. We selected the following ROI: anterior cingulate cortex (ACC), amygdala, anterior insula, orbitofrontal cortex (OFC), dorsolateral prefrontal cortex (dlPFC), dorsomedial prefrontal cortex (dmPFC), ventrolateral prefrontal cortex (vlPFC) and ventromedial prefrontal cortex (vmPFC).

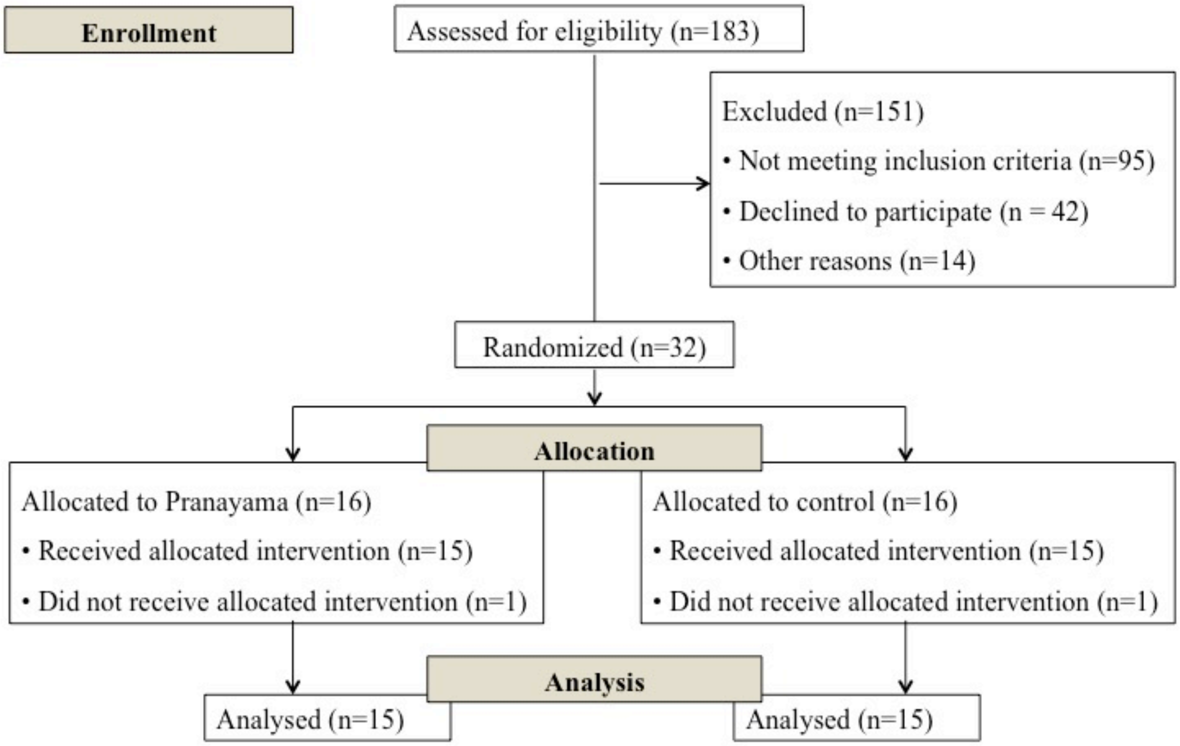

Figure S3. Trial profile.

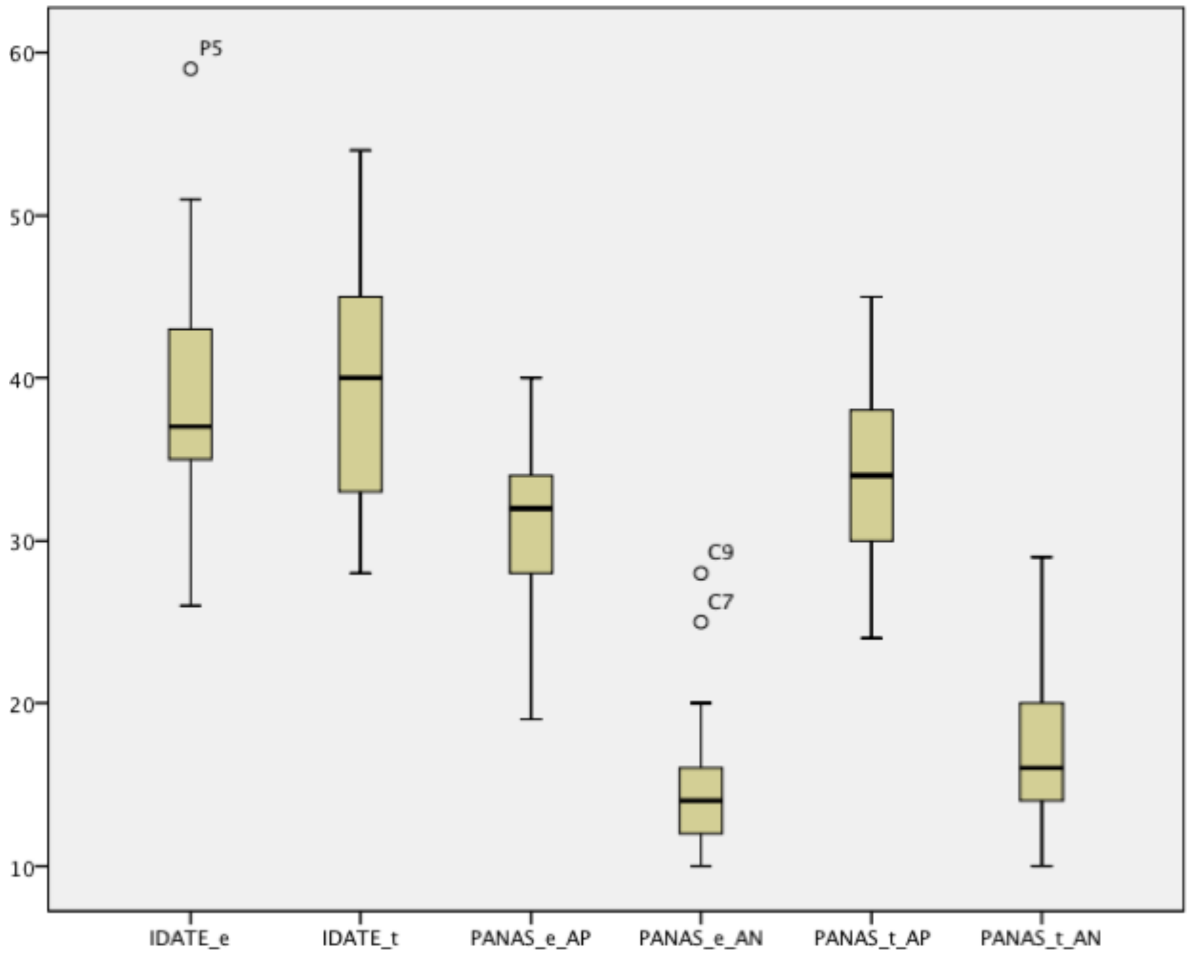

Figure S4. Boxplots for outlier identification applied to STAI and PANAS scales. The analysis identified one outlier from the *pranayama* group in state anxiety and two others from the control group in state negative affect. We considered outliers as values above or below 1.5 times the interquartile range.

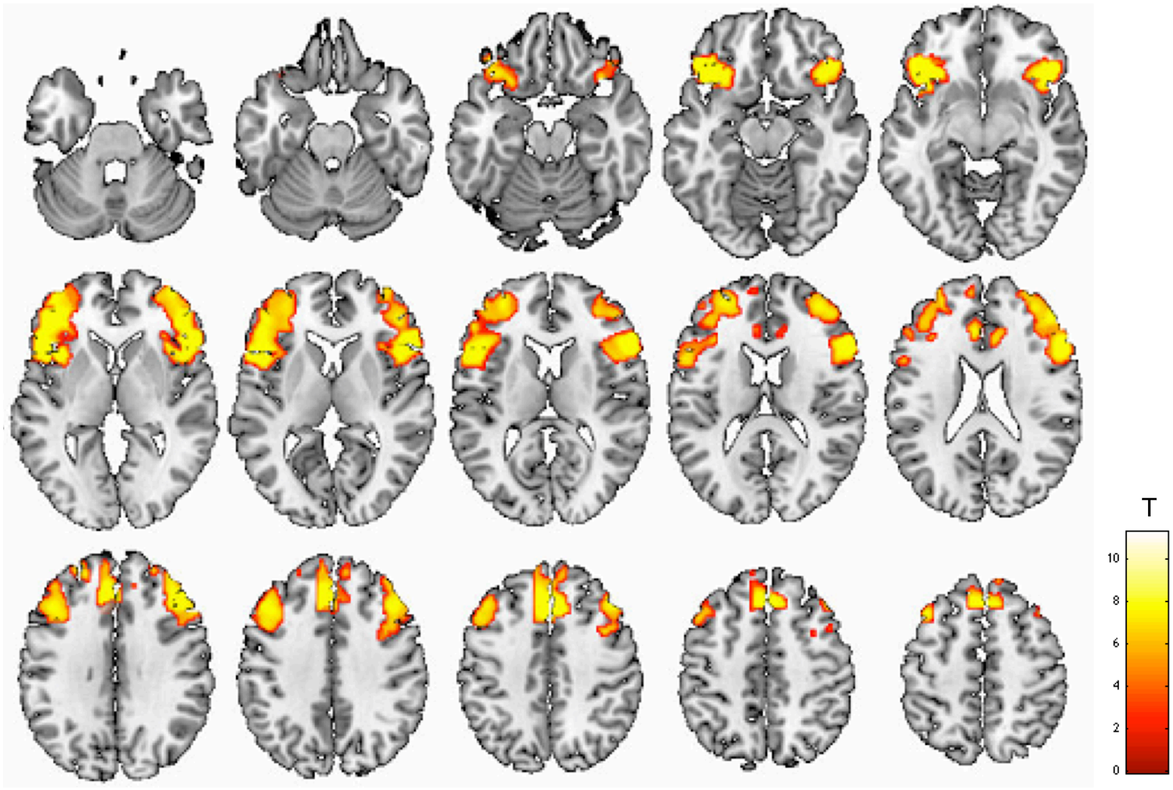

Figure S5. Baseline main effect of the task in the NEG condition. Statistical maps obtained from data of all the volunteers (N = 26) before the intervention. We used p_{cdt} <0.001, small volume correction, and p_{fwe} <0.05.

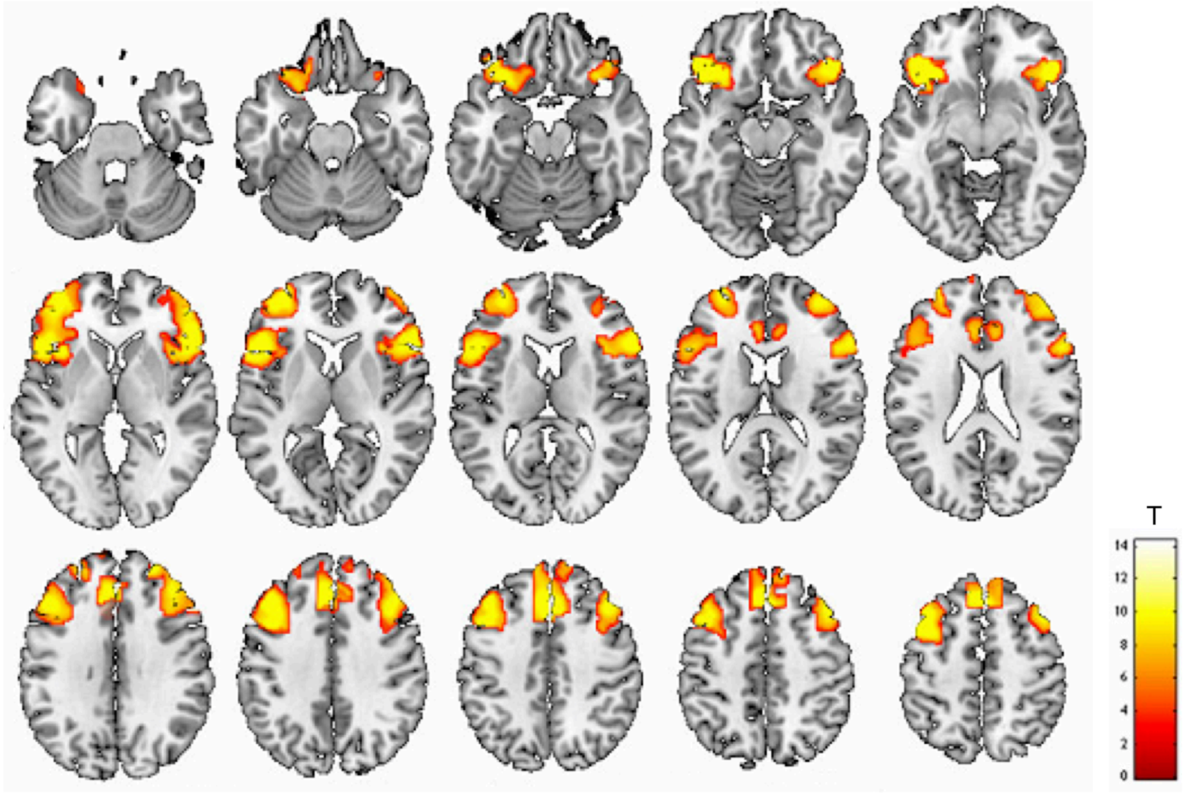

Figure S6. Baseline main effect of the task in the REAP condition. Statistical maps obtained from data of all the volunteers (N = 26) before the intervention. We used p_{cdt} <0.001, small volume correction, and p_{fwe} <0.05.
